# Extending Participatory Sensing to Personal Exposure Using Microscopic Land Use Regression Models

**DOI:** 10.3390/ijerph14060586

**Published:** 2017-05-31

**Authors:** Luc Dekoninck, Dick Botteldooren, Luc Int Panis

**Affiliations:** 1 Information Technology, Research Group WAVES, Ghent University, 9052 Ghent, Belgium; dick.botteldooren@ugent.be; 2 Vlaamse Instelling voor Technologisch Onderzoek (VITO), Boeretang 200, 2400 Mol, Belgium; luc.intpanis@vito.be; 3 Traffic Research Institute, Hasselt University, 3500 Diepenbeek, Belgium

**Keywords:** personal exposure, health, policy, black carbon, noise, spatiotemporal models, air pollution, activity

## Abstract

Personal exposure is sensitive to the personal features and behavior of the individual, and including interpersonal variability will improve the health and quality of life evaluations. Participatory sensing assesses the spatial and temporal variability of environmental indicators and is used to quantify this interpersonal variability. Transferring the participatory sensing information to a specific study population is a basic requirement for epidemiological studies in the near future. We propose a methodology to reduce the void between participatory sensing and health research. Instantaneous microscopic land-use regression modeling (µLUR) is an innovative approach. Data science techniques extract the activity-specific and route-sensitive spatiotemporal variability from the data. A data workflow to prepare and apply µLUR models to any mobile population is presented. The µLUR technique and data workflow are illustrated with models for exposure to traffic related Black Carbon. The example µLURs are available for three micro-environments; bicycle, in-vehicle, and indoor. Instantaneous noise assessments supply instantaneous traffic information to the µLURs. The activity specific models are combined into an instantaneous personal exposure model for Black Carbon. An independent external validation reached a correlation of 0.65. The µLURs can be applied to simulated behavioral patterns of individuals in epidemiological cohorts for advanced health and policy research.

## 1. Introduction

In recent literature, the investigation of the health effects due to multiple simultaneous exposures to environmental stressors is referred to as the “eco-exposome”. It is the sum of all environmental burdens to which a person is exposed and its main purpose is to address, interpret, and disentangle the health effects of the different stressors. The National Research Council committee (NRC) of the National Academy of Sciences compiled two sets of recommendations regarding the vision and strategy for the exposome [1]. The first set of recommendations addresses the more general concerns on the highly multidisciplinary nature of the exposure field and expresses the need to align research from many different fields. It also reaches out to the policy domain. The research should result in better support of regulatory and societal challenges. The scientific efforts have to result in better human and ecosystem protection. The document emphasizes strongly the need for interaction between personal exposure, external dose, internal dose, and the toxicology pathways of the pollutants. The gathering of data in this field through participatory sensing campaigns and mobile measurements is expensive and requires a lot of technical support. It is therefore difficult to achieve large and representative population samples. The difficulty to extrapolate the exposure measurements to representative populations for epidemiological research and governmental policy is recognized as the most important issue to be solved in exposome research [1].

The second set of recommendations the NRC committee sums up are more detailed concerns on the interactions between several components in the exposure data. It acknowledges the importance of personal time-activity patterns, activity related dose corrections, the potential effects of behavioral changes, and several feedback mechanisms. The explicit inclusion of the upstream forcers to connect to the policy support is on the wish list as well [1].

In another review “Integrating health and environmental impact”, a modified ecosystem enriched Drivers, Pressures, State, Exposure, Effects, and Actions or “eDPSEEA” framework is proposed, addressing similar concerns due to the “continuing failure to truly integrate human health and environmental impact analysis” [2]. Many of the data flow properties of the eDPSEEA approach match the recommendations of the NRC committee documents, and it explicitly expresses the need to extend the conceptual framework to include “an effective and robust science-policy interface”. In a sense, the approach of the eDPSEEA framework is a larger scale and economic-indicators-driven vision for environmental evaluations and enhanced policy support, while the eco-exposome builds on the biomedical indicators, biomarkers, and toxicological parameters. Both are expressing the same concerns.

Other reviews focus on good practices for the actual personal exposure measurement campaigns that will feed the eco-exposome research. A state of the art overview of current practice in participatory sensing measurements is presented by Steinle and colleagues, evaluating numerous “must-have” properties of the research set-ups [3]. One of the basic concepts is the monitoring of specific micro-environments, enabling detailed modelling of the micro-environment specific spatial and temporal variability.

Taking all this information into account, the common concern of these authors is that the exposure scientists, in a multi-disciplinary context, do not provide models that can predict real-life exposure, including activity-based interpersonal variability. In this publication, we present a generalized methodology to model and extrapolate participatory sensing data towards the personal exposure of any mobile population. This is the summary of the PhD of Luc Dekoninck [4]. The methodology itself was not the primary goal of the PhD but emerged from the technical solution of the self-imposed validation approach: ”Can we predict external participatory sensing data by extracting knowledge in an extreme spatiotemporal resolution from real-life micro-environment specific measurements?”

In the specific context of exposure to traffic-related particulate matter, the main restriction in the models is the lack of knowledge of the spatiotemporal variability of the traffic and traffic dynamics. In the PhD, the traffic information is retrieved from simultaneous noise measurements. The scientific support given to improving the Quality of Life and Health assessments using noise as a proxy emerged from the prior work of the authors [5]. A multi-level indicator set predicts the subjective response of a Quality of Life survey (QoL). The calculated noise levels at the facade combined with the simulated noise levels along the first 300 m of roads near the dwelling are the strongest components in the QoL-model. The main goal of the PhD was to move from simulated noise levels to measured noise levels and to use this information to improve the exposure predictions for traffic-related particulate matter (Black carbon and Ultrafine Particles (UFP). These models will be summarized in this publication as a proof of concept of the presented methodology (Section 3). Noise as a proxy for traffic is not the focus of this article but an example case of attributing the participatory sensing data in a high spatiotemporal resolution. Several authors explicitly refer to the use of ubiquitous data gathered in participatory campaigns [6,7]. Noise assessment is, in this approach, a high level type of ubiquitous data.

The methodology can be split into a general data workflow and the core functionality that we refer to as microscopic Land Use Regression (µLUR). First the concept of “µLUR” is explained (Section 2.1). It maps directly to the concerns listed by the referenced authors [1,2,3]. µLURs model the spatiotemporal behavior of the indicator based on participatory sensing data and deliver the actual prediction functions for the activity sensitive indicators. The data workflow covers the processing of the population data and the external data necessary to calculate the indicators on a personal level (Section 2.2 and Section 2.3). The data workflow can be applied to any mobile population with only one restriction; the underlying population dataset has to be attributed with an adequate set of personal information to enable the reconstruction of the typical commuting behavior and/or activity pattern of the individuals. The last step is the application phase for epidemiologists (Section 2.4) and for policy support (Section 2.5). It is the authors’ opinion that the presented methodology provides an answer to many concerns listed by the referenced authors [1,2,3].

## 2. Extending Participatory Sensing with Microscopic Land-Use Regression

### 2.1. Microscopic Land-Use Regression Modeling

Participatory sensing campaigns differ from standard “designed” scientific experiments in a few fundamental aspects. The most important difference is the uncontrolled sampling. The measurements cannot be constrained to boundary conditions as is common in designed experiments. This implies that the evaluation of the gathered data will be more complex, and, above all, it will be virtually impossible to gather an unbiased dataset. On the other hand, participatory sensing provides “real life” data not limited by the design of the experiment. A successful model based on the real life data will therefore be much more applicable to real life populations compared to the restricted results of the designed experiments. This requires models which explain as much variability as possible over all driving forces in the real life data. The spatiotemporal variability in the participatory sensing data can be extreme, which implies that the assessment of the driving forces also requires a similar spatiotemporal resolution. The prerequisite of the approach is to conserve the variability and hence only perform a low level of aggregation and/or smoothing on the raw measurement data. A critical point is the availability of external data to attribute all relevant driving forces of the indicator.

Bias in the measurement campaign is the next concern. The answer in this methodology is to sample explicitly all relevant dimensions. In the specific case of air pollution exposure, this implies sampling all meteorological situations, seasonal variability, and traffic situations. The model should be able to explain this variability and, by doing so, resolve the main risk factors responsible for the unquantifiable bias in designed campaigns. This will be addressed in detail in the discussion.

Exposure to environmental burdens is frequently investigated on the aggregation level of the activity related type, purpose, or micro-environment since the average value of the indicator is highly linked to the properties of the micro-environment. Investigating the instantaneous behavior of the indicator within each micro-environment or activity type will split the data into logical and manageable datasets. Building activity or micro-environment specific models is therefore the preferred method. Understanding the spatiotemporal behavior of the indicator within a single activity will be the key to understanding and extrapolating the activity based exposure. Each type of activity will require a spatiotemporal resolution matching the expected variability during the activity. The spatiotemporal resolution will be related to the micro-environment in which the activity takes place.

The activity becomes a spatiotemporal object with a set of standardized attributes: the episode, the purpose, the micro-environment, and the location. The location can be a fixed position or a travelled route. Within the activity specific models, the specific driving forces of the indicator have to be retrieved from available external data or a simultaneous assessment of potential driving forces in the participatory campaigns. Many of the driving forces or, more specifically, the underlying external data to introduce the driving forces into the model will express non-linear behavior towards the investigated indicator. Developing a successful predictive model will thus require non-linear modelling techniques. The potential for the non-linear modelling is intertwined with the low aggregation level of the measurement data and the spatiotemporal resolution of the external data.

This described trajectory from participatory data to predictive model is referred to as microscopic and micro-environment specific non-linear spatiotemporal land-use regression modelling (µLUR). The features of the methodology are summarized in Table 1.

**Figure 1:**
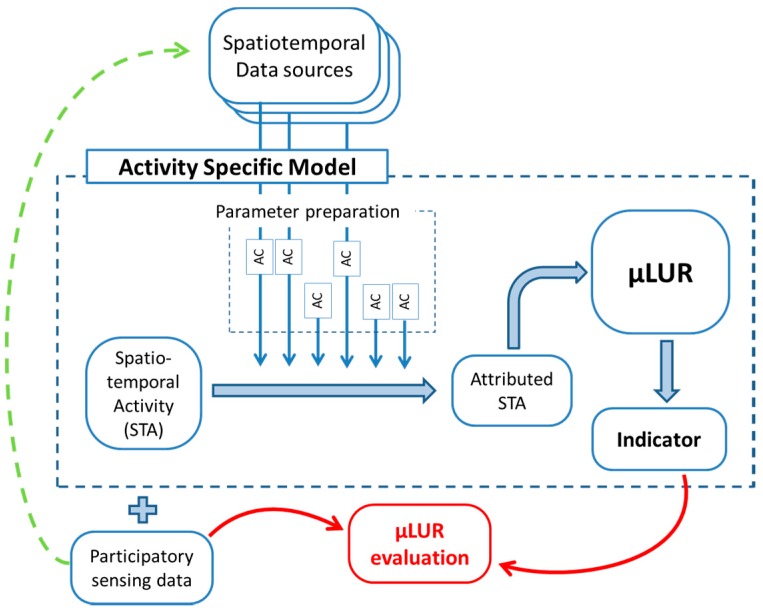
The activity specific model combines the spatiotemporal attribution of the activity and the µLUR model. The activity calculations (AC) convert the external data to activity specific attributes (blue arrows). The red arrows represent the µLUR validation process. The ubiquitous information gathered in parallel with the indicator can be used as external data (dashed green arrow).

The µLUR model does not exist on its own but includes the specific features of the external data and matching pre-processing of the external data. This is visualized in Figure 1. Each model covariate is calculated from one or more external datasets with specific properties, shortcomings, and data preparation. Converting the raw external data into a high quality covariate is part of the investigative trajectory for each activity specific model, visualized as Activity Calculations (AC). The combination of the external data pre-processing and the µLUR itself is referred to as the “Activity Specific Model” (ASM). The aim is to achieve an activity specific model transferable to other population datasets. This aim can restrict the model covariates to general and/or publicly available data (see Discussion).

The participatory sensing data is split into the relevant activities. For each relevant type of activity, a separate ASM is built. In Figure 1, the structure of a single ASM is presented. The ASM includes the parameter preparation based on available external data sources. The spatiotemporal data series of the activity are attributed in space and time. The µLUR is evaluated by comparing the predicted value with the indicator measurement.

### 2.2. Activity Based Mobile Populations

**Figure 2:**
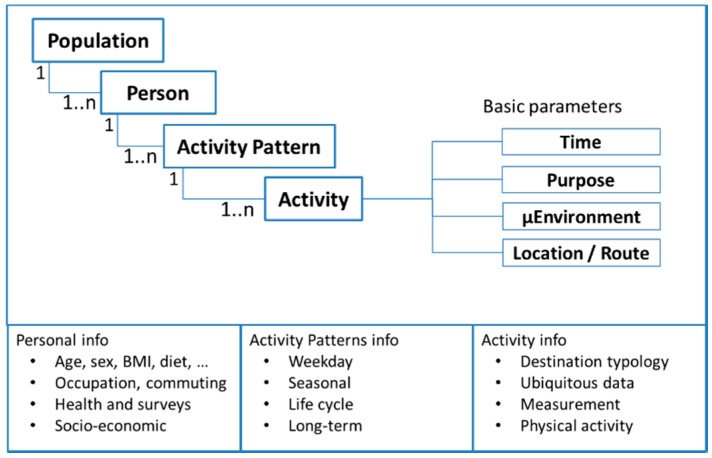
Personal objects structure and examples of the objects attribution.

The individuals in a population perform activities at fixed locations or while being mobile. To calculate activity specific indicators, the breakdown of the data into individual behavior has to be performed up to the detail of the activity. The person related objects split up and cascade down into three levels; Person, Activity Pattern, and Activity (Figure 2). The Activity is the link between the activity specific model and the individual. The Activity Pattern is in most cases a typical daily pattern of the Person. Within the activity, a temporal resolution is chosen, matching the expected temporal variability of the investigated indicator. The matching µLUR will be built with a similar spatiotemporal resolution. Different activity types within the Activity Pattern can have different temporal resolutions.

The interaction between Person characteristics and indicator is not restricted to the activity properties. Any parameter of the Activity Pattern and Person can become a relevant parameter in the activity specific model. As an example, the socio-economic status is linked to the quality of the ventilation of the dwelling which can influence the indoor exposure to an outdoor pollution source. A personal parameter “active smokers in the household” can interact with the indicator as well. Including these parameters in the µLURs is a multidisciplinary decision for each project-specific application. Standardizing the relevant personal and activity specific attributes in the population databases will improve transferability across populations and health studies.

### 2.3. Personal Exposure Data Workflow

**Figure 3:**
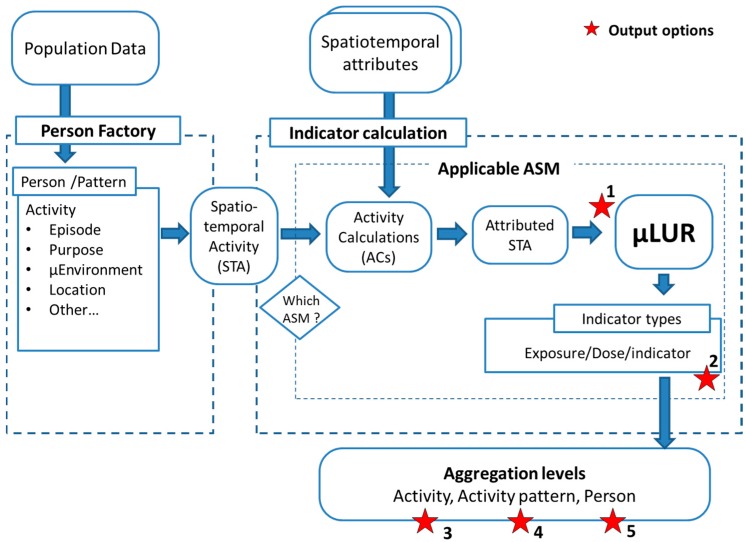
The person factory creates the person, time-activity pattern, and activity objects from the external population data. The first action in the “Indicator Calculation” is to dispatch the activity to the applicable activity specific model (ASM). The Spatiotemporal Activity assembles all personal data and triggers the indicator calculation. The applicable ASM contains a sequence of Activity Calculations retrieving the external data and performs every relevant action on the data (external and activity data). This results in an attributed Spatiotemporal Activity. The µLUR is applied and results in the indicator. The five stars identify the stages in the process where data can be retrieved and reporting is initiated.

The Personal Exposure data workflow merges the mobile population and the indicator calculation into an operational methodology. The activity based Indicator Calculation combines a set of activity specific models to cover the required types of activities for the targeted indicator (Figure 3). The Person Factory is a toolbox converting population and person data into the activity based mobile population. Indicator Calculation and Person Factory have the central object in common; the activity with its spatiotemporal information (STA). The first action of the Indicator Calculation is to dispatch the activity to its matching activity specific model.

From the perspective of the exposome concept, any relevant indicator calculation can be added and can be processed on the same population dataset. Merging and evaluating multiple indicators is project specific and is not covered within the scope of this research article.

The data workflow has different output options matching the different phases of the research project (visualized with stars in Figure 3). The first phase is the processing of the participatory sensing data and the µLUR development. In this phase, the participatory sensing itself is used as the population data. The tracked people, their attributes, their location data, the acquired measurements, and other ubiquitous information are used to create spatiotemporal activities (STA). In this stage, the data will be supplied and processed in the chunks matching the strongest indicator discriminator; in practice, this will be activity and/or micro-environment (bicycle data, in-vehicle data, indoor leisure at home, indoor cooking, etc.) to build the activity specific µLURs. In each activity specific model, all potential relevant external data are mapped in space and time at the relevant spatiotemporal resolution for that specific type of activity. The µLUR is the unknown component. At the “output point 1”, the dataset on which the actual µLUR is developed is available. The aim of the µLUR is to provide a prediction for the indicator for each time step during the activity. Any modelling technique or function can fill in the µLUR. In the presented case (see Section 3), Generalized Additive Models (gam) are used in the µLUR.

In a second phase, an external validation can be performed with the same data workflow. An independent participatory sensing dataset measuring the same indicator is used to create persons, activity patterns, and activities with the person factory. The indicator calculation attributes the activities in an identical way as defined in the applicable ASMs in the first phase. The µLUR provides a prediction for the indicator. Note that the temporal resolution of the indicator measurements in the validation campaign does not have to match the temporal resolution of the ASMs as long as the spatiotemporal activity can be provided in that temporal resolution. The resolution of the available data and the project setup will designate the appropriate aggregation level to perform and report the external validation. The validation level can be the temporal resolution of the ASM at output point 2, but performing the validation on the aggregated levels of activity, activity pattern, or person is also a valid option (output 3, 4, and 5).

### 2.4. Personal Exposure for Epidemiologists

The participatory campaigns used for the µLUR building are typically not performed on the subjects in the available epidemiological cohorts. In selected smaller studies, tracking data for the subjects can be available. In both cases, the data workflow and the µLURs can be applied, but there is a critical restriction. The survey information on the subjects has to provide enough detail to reconstruct the activity pattern in the temporal and spatial resolution of the activity specific models. For studies on traffic related health effects, the personal information should at least include the dwelling and workplace location, work regime, and modal choice. This allows the reconstruction of a diurnal pattern and the episodes and duration of the in-traffic activities using network based geographic information solutions. In smaller studies, tracking data can be used to feed the Person Factory.

An interesting aspect of the proposed methodology in this type of application is the reusability of the personal activity patterns of the subjects in the epidemiological dataset. The mobility behavior of the subjects becomes a standard feature that is available for all disciplines to implement Indicator Calculations. This links immediately to the exposome concept; different disciplines add their specific knowledge through discipline specific Indicator Calculations. Discipline or exposure of specific personal attributes can be added for specific indicators. When a personal attribute proves relevant at the population level in a specific study, it should be considered to assess the attribute in other studies as well. Each attribute becomes a potential confounder when comparing health indicators and health outcomes across disciplines.

### 2.5. Personal Exposure for Policy Makers

Another application fits in the need to provide effective and robust science-policy interfaces (see Section 1). The indicator calculations were validated in the previous phases and can now be used in the policy field. The spatial resolution of the µLUR can be extremely high, and the methodology is micro-environment and route sensitive. This fits the spatial and the temporal resolution of the policy questions at the level of local governmental entities. The indicator calculations are sensitive to modal choices and to improved (local) traffic network functionality. In most cases, the policy scenarios will be initiated by modifying the composition of the population feeding the Person Factory. Modal choices can be changed, and exposure features along the trajectories can be used to divert trips to lower exposure. Dwellings and destinations can be modified to assess urban development options and demographic changes. The driving forces of the µLUR can be impacted by the scenarios as well, but this will require expert decisions on long-term changes for the driving forces.

The potential changes in the behavioural patterns of the persons in the study population become the main variable in the policy applications. The changing population characteristics are provided as a part of the external data by the mobility and urban planning disciplines. The data workflow and validated µLURs provide highly detailed information on a set of relevant indicators to the policy makers.

## 3. Example Case: Personal Exposure to Traffic Related Black Carbon

A summary of the participatory campaigns and the resulting micro-environment specific µLURs is presented in this section. It is not the aim of this publication to be exhaustive on all details of the presented models. Details can be found in [4].

In this specific case, the most important entangled driving forces of the personal exposure are the traffic, the influence of instantaneous meteorological conditions, and the changing background concentration throughout the seasons. The models predict external exposure to Black Carbon (BC); activity related dose corrections are explicitly not included. Our aim is to validate the external exposure assessments only. Dose corrections are relevant when the health evaluation is part of the outcome.

An independent external validation is available and is based on a participatory sensing campaign performed by Vlaamse Instelling voor Technologisch Onderzoek (VITO) (Flemish Institute of Technological Research) in 2010 and 2011 [8,9]. This participatory campaign tracked 31 couples for one week each across Flanders. The activity diary is recorded, Black Carbon is measured with a mobile device at a temporal resolution of 5 min, and the in-traffic activities are tracked with the Global Positioning System (GPS). This dataset is converted in an activity-based mobile population according to Section 2.2. The aim is to predict the instantaneous Black Carbon exposure for each person-day, covering all activities, including the in-traffic exposure. This validation method is a unique way to verify that the models predict real-life exposure.

### 3.1. µLURs for Bicycle, In-Vehicle and Indoor Exposure

#### 3.1.1. The Instantaneous µLUR for Cyclists

The µLUR model for cyclists is based on 220 h of simultaneous measurements of Black Carbon and noise while commuting in the rush hour [10]. An international validation is available and also extends the technique to traffic related UFP [11]. The models are instantaneous models, predicting the traffic related exposure for a specific location of the bicyclist for the instantaneous meteorological conditions and the background concentration from a continuous air pollution measurement station. The instantaneous traffic information is retrieved from noise measurements.

The noise measurements include the spectral content of the noise exposure, which allows the detection of the traffic dynamics of the local traffic flow [10] (visualized in chapter 5.3.2.1 in [4]). The spectral content of the noise measurements can distinguish between engine related noise and tire related rolling noise. This information quantifies the traffic dynamics. The BC emission of the vehicles is a strong function of the engine regime. This physical relation ‘engine noise’ to ‘BC emission’ is a crucial property of this model. The low frequency noise is a combined measure of the number of vehicles, the acceleration of the traffic flow, and the distance to the source of the bicyclist. The high frequency noise is an indirect measure of the speed of the traffic flow. The external covariates and the Black Carbon exposure are evaluated in a temporal resolution of 10 s. With a typical speed of 18 km/h of the cyclists, this results in a model with a spatial resolution of 50 m along the road segments with a built-in correction of the relative position of the bicyclist towards the traffic lanes.

**Figure 4:**
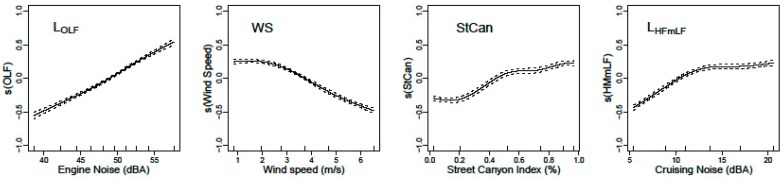
Splines of the BC_local_ generalized additive model (gam). The dashed lines are the confidence interval of the splines.

The fundamental improvement is the capability to disentangle the variability in the exposure measurements into the local variation due to the instantaneous local traffic exposure, the instantaneous meteorological influences (wind speed), and large scale seasonal influences on the emission, dispersion, and potential accumulation of the background levels. The instantaneous exposure BC_total_ is separated in a background component BC_bkg_ and local component BC_local_ (see Equation (1)). The local component is resolved with a generalized additive model (see Equation (2)). The dominant parameters in the local component are the engine related noise L_OLF_ and the wind speed (WS). The influence of the street canyon features (Street Canyon Index StCan) and speed related attribute L_HFmLF_ are statistically significant but weaker parameters. The splines of the gam model are shown in Figure 4.

BC_total_ = BC_bgk_ + BC_local_ (1)

Ln(BC_local_) = gam(L_OLF_, WS, StCan, L_HFmLF_) (2)

An extension on this model extrapolates the instantaneous model to a variant providing yearly average exposure along the cyclists’ trajectories by applying the instantaneous model to a full year of meteorological conditions [12].

#### 3.1.2. In-Vehicle Exposure

The µLUR model for in-vehicle is based 165 h of Black Carbon measurements by nine volunteers performing real-life trips over the year 2013. The details of this model can be found in chapter 4.5 in [4]. The only design in the measurement campaign was to exclude participants that smoke. The vehicle trips are part of the daily behavior of the volunteers. The exposure inside the vehicle is evaluated at a temporal resolution of 10 s. The main challenge of the model is to find and quantify the variables expressing the short term variation of the in-vehicle exposure. The typical speed of a car is between 10 and 120 km/h, depending on the road and local traffic situation. This results in a dynamic spatial resolution between 25 and 300 m.

The in-vehicle Black Carbon concentration is a function of the distance to and properties of the preceding vehicles, the density, and the traffic dynamics of the preceding vehicles but also ventilation settings and other parameters that cannot be captured in a voluntary participatory sensing campaign. The only alternative is to select a set of spatiotemporal parameters that can explain at least a part of these driving forces in an indirect stochastic approach.

**Figure 5:**
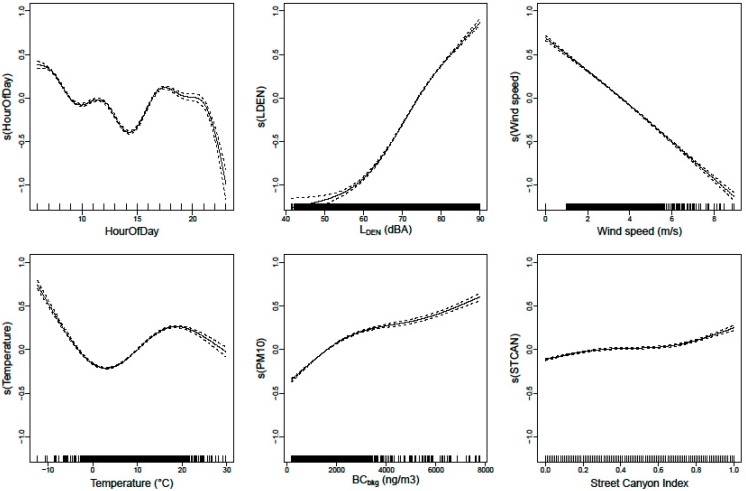
Splines of the gam model for the six covariates; the diurnal pattern in the Hour of Day covariate, the diurnal patterns of meteorological aspects in particulate matter dispersion and coagulation, and the dynamics of the engine emission as a function of the changing traffic dynamics over de diurnal pattern.

Traffic data is added to this Black Carbon µLUR in a direct way, using external traffic data and compared with an indirect traffic assessment by retrieving the L_DEN_ noise level from a noise map. The vehicle tracks are matched to the road segments using a custom spatial procedure. This procedure removes the spatial errors in the GPS tracking and improves the quality of the in-traffic attributes. The noise map approach results in a better model compared to the traffic data approach. This improvement could be due to the fact that the noise map combines measures of heavy duty vehicles and cars in one single value. The noise map also includes a built-in combination of multiple sources at crossings, indirectly expressing the changing traffic dynamics. It results in a better prediction of the highest exposure values for the dynamic traffic conditions when complex interactions of vehicle flows occur. For the final model, six covariates (Hour of Day, L_DEN_ noise map, wind speed, temperature, background exposure (BC_bkg_), and Street Canyon Index (StCan)) are selected to build the in-vehicle µLUR (see Figure 5).

The hour of the day fits the diurnal pattern of in-vehicle exposure, merging all diurnal patterns influencing the in-vehicle exposure into a single covariate. More information on this approach is available in the discussion (see chapter 4.3). The outdoor temperature covariate is reflecting the changes in ventilation settings of the car drivers. The correlation between the measured exposure by trip in the external validation data and the model prediction of the exposure for these trips reaches 0.65.

#### 3.1.3. Extrapolation of the µLUR for Cyclists with Noise Maps

**Figure 6:**
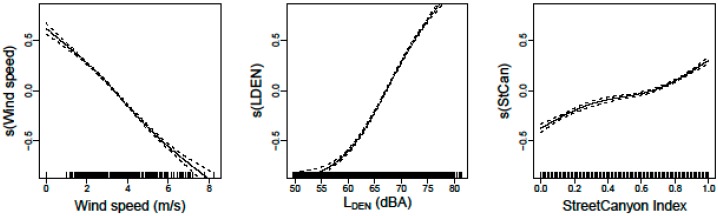
Splines of the extrapolated variant of the instantaneous bicyclist exposure model using a L_DEN_ noise map as the underlying alternative traffic layer.

The instantaneous bicycle model is restricted to the mapped roads in the city of Ghent (Belgium), but the external validation campaign covers the whole region of Flanders. Since the mobile noise measurements are not available for Flanders, a spatial extrapolation of the instantaneous bicycle model is required. To achieve this, the noise measurements are replaced by the noise map (same map as the in-vehicle model). The spatial and temporal quality of the traffic characterization is strongly reduced by this action. The noise measurement based traffic information, available on all trajectories in the instantaneous model, is replaced with a dataset in which traffic information is only available on the main roads and local connecting roads matching the official Flemish traffic model. The effect of this reduction of traffic quality on the models is not within the scope of this research article. The resulting model is still an instantaneous model providing a prediction of the exposure of the bicyclist. Since no spectral information including the traffic dynamics is available, the two noise covariates, L_OLF_ and L_HFmLF_, are replaced by a single covariate (L_DEN_) (see Figure 6).

#### 3.1.4. At Home Indoor Exposure

The last activity to be modelled is the indoor exposure. It builds upon the extrapolated bicycle model. The lowest temporal detail of the external data in the indoor model is 30 min. The temporal resolution of the µLUR is set to the temporal resolution of the validation campaign (5 min). The basic assumption is that the cyclists sampled the Black Carbon exposure at random over the whole exposure surface. This neglects the sampling restriction of the cyclists (sampling along road network). The rationale behind this hypothesis is based on the observation that the distance-to-source dependence of air pollution and noise pollution is similar, e.g., both are a function of 1/r^2^. This implies that the spatial features of the noise map are a valid proxy for the air pollution dispersion models. In the used noise map, the noise sources are included in a spatial detail of meters sensitive to the actual physical position of all roads in the Flemish region. In many cases, the idealized link of the underlying traffic model is used for maps of this spatial extent. The noise map acts therefore as a “distance to source weighted” external data layer with a higher local precision when compared to standard (large scale) dispersion models. Even more important, this spatial layer provides a single covariate with traffic data for the µLUR, while, in the classical approach, this information is provided by stepwise buffers, which are by design less sensitive to the strong distance to source effects of particulate mattter exposure [13,14].

The independent local and the background component of the cyclists’ Black Carbon exposure model extend into an indoor model. The background component expresses large scale meteorological and seasonal effects. It is implemented as the time series of a representative background measurement location (identical to the bicycle model) but adjusted with a large scale spatial correction to provide a solution for the whole region (Flanders, Belgium).

The function to convert the noise map into the local component of the traffic related exposure is available in the extrapolated variant of the bicycle model. The bicycle model is restricted to rush hour. To provide a diurnal solution, a fixed diurnal correction is applied to the L_DEN_ map to provide estimates for every hour of the day. The model hypothesis is summarized in Equations (3) to (5). The bicycle model provides outdoor exposure estimates, and the indoor model requires an outdoor to indoor correction. No independent temperature dependency of the indoor to outdoor ratio (I/O ratio) was available for indoor BC exposure. To resolve this missing information, a temperature dependent outdoor to indoor correction is fitted to match the prediction to indoor measurements. This resulting fitted correction is within the range of available literature for PM2.5 indoor-outdoor ratios (see Equation (6)).

BC_indoor_ = (BC_bkg,outd_ + BC_loc,outd_). Out_Incor_(T_day_) (3)

L_DEN,T_ (p, t) = L_DEN_(p) + Traf_diurn,cor,dB_ (hour_of_day(t)) (4)

BC_loc,outd_(p, t) = gam_loc,noisemap_(L_DEN_(p, t), WS(p, t), StCan(p)) (5)

Out_Incor_(T_day_) = 1/(1.29 − 0.018 T_day_) OutIn_const_ (6)

The temperature and BC background concentration show inverse relations for the indoor exposure. Applying the temperature based I/O ratio in the indoor BC model resolves the inverse relation of the background exposure. This illustrates the validity of the I/O correction. In this modelling approach, the two temperature dependent components of the indoor exposure are disentangled. All details are available in chapter 4.6.5 in [4]. We are aware of the limitations of the noise map as the extrapolation layer of this model. A pilot experiment to use the µLUR technique at dwelling facades illustrates the potential for the future improvements (see chapters 4.7 and 6.1 in [4]).

### 3.2. Personal Exposure to Black Carbon

The prediction of personal exposure is a combination of the presented activity specific models, applied to the entire time activity pattern of the individual in the population dataset. The validation is achieved by feeding the person factory with the time activity patterns gathered in the independent Black Carbon exposure campaign [8,9]. For each activity, the matching activity specific model is applied. However, a specific model is not available for all micro-environments, and some additional assumptions are added. Pedestrian exposure is assessed with the bicycle model. Public transport is assessed with the in-vehicle model. The very limited number of railway activities is mapped to the bicycle model, but, as road traffic noise levels at railroads are generally very low, the contribution to diurnal exposure often remains limited. Exposure at the workplace is predicted with the at home indoor model due to lack of external workplace specific Black Carbon exposure data. The discrepancies at the workplace are evaluated by the purpose of the activity, and the discrepancies match the qualitative expectations. Activities with a high potential of non-traffic related BC exposure are systematically underestimated (see chapter 5.2.4.2 in [4]).

**Figure 7:**
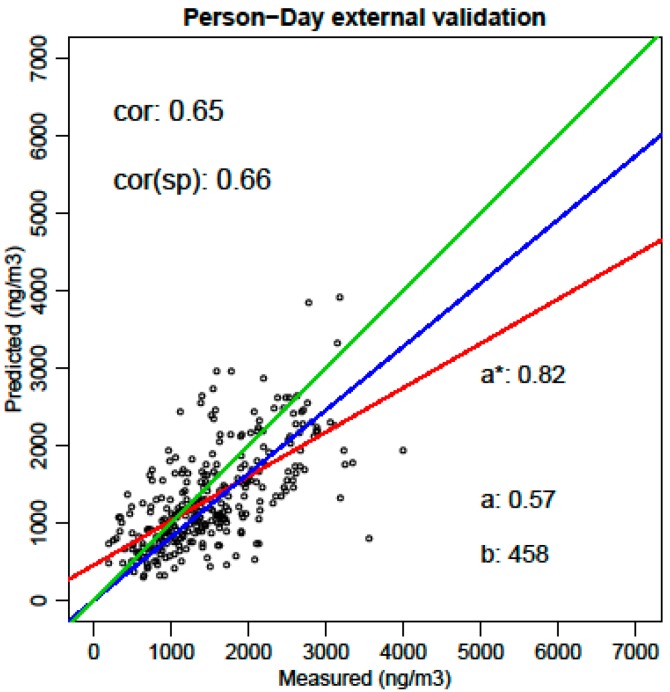
External validation (prediction versus measurements) of the personal daily instantaneous exposure for Black Carbon including in-traffic exposure using the µLUR methodology (293 person-days). Regression lines forced through zero (blue) and free regression (red) are compared to the ideal case (green).

In Figure 7, the external validation of the diurnal personal exposure is presented. The evaluation level in this case is the activity pattern for a single day (option 4 in Figure 3) since the sensitivity of the instantaneous models has to be validated for the combined variation of the instantaneous background concentration and meteorology of the specific day and in-traffic specific behavior of that person on that specific day. An overall correlation of 0.65 is achieved. This is considered to be a valid personal exposure model. A more detailed discussion on the quality of the validation by micro-environment and by purpose of the activities can be found in chapters 5.2.3 and 5.2.4 of [4].

## 4. Discussion

### 4.1. Moving from LUR and µLUR

Land-use regression based on participatory sensing data is a big data-like method searching for indirect spatial or spatiotemporal correlations between external data and the indicator under investigation [13]. The parameters of the LURs correlate with the investigated indicator without explicit physical and chemical relations. A LUR does not attempt to provide an analytical function between parameters and outcome; it only attempts to transfer the spatial features of the external data to the indicator at hand and is expected to provide a valid prediction.

The µLURs use the same underlying principle, transferring spatiotemporal external information to the indicator, but the focus is more on physical correlations with the driving forces. Due to the increased spatiotemporal resolution, more potentially relevant information can be retrieved from the measurements. The high resolution µLUR covariates can be supported by scientific quantifiable knowledge if external data can attribute the driving forces in the required spatiotemporal resolution. In the presented case, the noise emission of vehicles and its spectral composition is the key. It provides external data on the driving forces of the spatial variability of the emission of particulate matter. This is due to the physical relationship; “engine regime determines engine noise as well as particulate matter emission”. The knowledge of the noise emission of vehicles adds value to the air pollution discipline currently not available by any other technology or data source. This multidisciplinary aspect is crucial. Merging knowledge can provide the missing data in the adjacent disciplines. The in-vehicle model shows that increasing the temporal resolution, even without instantaneous traffic information, can also result in a valid model. Increasing the temporal resolution and attributing the measurements in that increased resolution is the fundamental step when moving from LUR and µLUR.

This also illustrates that the µLUR features in Table 1 are not binary evaluations. The bicycle model based on instantaneous noise measurements ranks higher for feature 2 compared to the in-vehicle model and indoor model. The city-wide noise mapping methodology in [12] illustrates that noise measurements can provide the required proxy for the traffic data without sampling throughout the seasons (four passages quantify the traffic adequately and the mobile noise measurement is not sensitive to season or weather conditions). Thus short-term noise measurements provide sufficient information on the spatial variability of the driving force with full area coverage. The next step is improving the noise maps by including the city-wide noise based traffic proxy. The quality of the in-vehicle model and the indoor model will increase accordingly.

### 4.2. Personal Exposure and µLURs, a Data Driven Solution

The combined methodology, µLUR, and the personal exposure data workflow have one important feature in common; ‘keep at all times as much of the variability in every aspect of the indicator and underlying external data in the application’. This is expressed in the population factory by acknowledging all personal variability up to the spatiotemporal behavior within a single activity, including micro-environment, origin, destination, and route choice. In the indicator calculation, this feature is achieved by building activity specific models in a high spatiotemporal resolution, preferably including spatiotemporal covariates with a quantifiable relation towards the investigated indicator. The external data has to provide information in a similar spatiotemporal resolution as required for the indicator and activity type at hand. The µLUR has to provide a valid prediction over all driving forces and other influencing factors. This requires scientific measurement campaigns and post-processing setups matching the first three features in Table 1. The complexity of the indicator calculation can be reduced by providing activity specific models (feature 4 in Table 1).

The complexity of the indicators implies the use of non-linear modeling techniques (feature 5 in Table 1). The underlying datasets have to be large enough before non-linear techniques can result in solutions that do not over-fit the data. Classical LUR are typically based on a few hundred data points and include multiple covariates, which are in many cases not fully independent (for example, traffic in multiple buffers and population density). These limitations of LUR inhibit the use of non-linear modelling techniques. µLURs are based on measurement campaigns of a similar magnitude but increase the temporal and spatial resolution due to the low aggregation level. The 128 h of data in the bicycle model are equivalent to 37,000 data points, the 225 h of in-vehicle data to almost 80,000 data points. When the driving forces can be quantified in this temporal resolution, the non-linear models become robust against over-fitting. Increasing the temporal resolution enables the use of non-linear modeling techniques by design. In the presented case, the fully independent external validation illustrated the transferability and validity of the models.

### 4.3. The Data Science Techniques in the µLURs

The “phase 1” µLURs is an instantaneous model with the fundamental functionality to disentangle the variability due to the actual driving forces and other disturbing factors. In the presented case, meteorology introduces important variability. The disentanglement does not need to be analytically complete over all known influences. If an external attribute is available that combines a number of influencing driving forces for which no analytical solution is available, an indirect data solution is an option as well. This is illustrated in the bicycle model. In the background adjusted solution for the cyclists, the background component is still a combination of several driving forces and meteorological effects. The seasonal variation of Black Carbon is mainly driven by changes in the emission of domestic heating. A small component might be attributed to seasonal variation in vehicle emissions (cold start and use of in-vehicle heating). The method cannot quantify nor investigate this in more detail. The seasonal aspects of meteorology leading to increased pollution levels (lower wind speed and more stable atmosphere in winter) are incorporated in the background measurement itself. Although it would be very interesting to understand these individual components, this information was not necessary to build a valid exposure model. The fundamental innovation and a direct effect of the instantaneous traffic assessment is the disentanglement of the exposure in a local traffic related component and a background component. It is the quantification of the local contribution in the measurements that results in models with a traffic related attribute as the strongest covariate.

A second example is available in the in-vehicle model. The background exposure is not a dominant covariate in the in-vehicle model, but a similar data science technique is important in this model. Several diurnal patterns affect the particulate matter exposure inside the cabin in a direct and indirect way. The diurnal exposure pattern is a combination of non-linear effects of different origin (scavenging, traffic counts and traffic dynamics during rush hour, particulate emission functions, wind, temperature, humidity, etc.). In the presented model, only one traffic related covariate is included as a daily average. The complex interaction of all diurnal patterns, including traffic and traffic dynamics, is resolved in a single ‘time of day’ covariate. These two examples illustrate the data driven approach very well.

### 4.4. The Benefits of Instantaneous Models

#### 4.4.1. Instantaneous and Yearly Exposure

For the investigation of long-term health effects, epidemiologists prefer yearly or long-term personal exposures. Understanding the instantaneous exposure enables the calculation of annual exposure with a new level of accuracy, as was shown in previous work [12]. Instantaneous models are, due to the physical and financial limits of participatory sensing campaigns, an indispensable step towards yearly personal exposure assessments [1,2,3]. It is important to notice the compatibility of instantaneous and long-term exposure in health research. Long-term health effects and short-term changes in in-body biomarkers can correlate with different variants of the µLURs. Correlations between short-term biomarkers and instantaneous exposure can reveal new research and policy options.

#### 4.4.2. Monitoring the Fast Evolving Driving Forces

Participatory sensing campaigns on large populations are not only very expensive but also require huge logistical and technical support. The standard requirement of participatory campaigns is to achieve a representative population. Participatory sensing is using new technology, and it depends on capable and willing volunteers. Reaching a representative population is virtually impossible, even without considering the many influencing and interacting factors of the investigated indicator. Understanding the variability within individual activities results in models that can predict exposure for similar activities of other individuals. This removes the requirement to assess a representative population in the participatory campaign. The required increase of measurements to quantify the seasonal influences is at least partially, if not completely, countered by reducing the need to sample representative populations. Data gathering can now focus on quantifying the variability over all driving forces. The population statistics, supplied through the person factory, extrapolate the personal assessments to valid population if the underlying µLUR explains the variability of the indicator over a sufficient set of relevant covariates. This combination of properties is the key to remove the bias from the data in the measurements.

This feature of the µLURs enables cost effective monitoring campaigns to quantify the evolution in the driving forces. In the case of traffic related air pollution, this is a combination of changing emission functions due to product related legislation, changing fleet compositions, and mobility behavior due to taxation measures. Proper assessment of the effectiveness of legislation is an important component in the presented “eDPSEEA” approach [2]. Once the driving forces are identified, monitoring can partially shift from a full assessment of the indicator towards the monitoring of the driving force itself. In the city-wide noise monitoring methodology, mobile noise measurements without Black Carbon or UFP assessments provide the driving force in the required spatial resolution and partial simultaneous measurements provide the data for the µLUR [12]. Together, the acquired data provides both the spatiotemporal driving forces and the function to extrapolate the results to long-term averages without losing spatial resolution.

### 4.5. Extensions into Epidemiology

#### 4.5.1. Multidisciplinary Aspects in Dose Assessments

Each discipline can define specific indicators, but multidisciplinary interactions within the indicators can be implemented. The complex and highly specific path of the multiple chemical components of air pollution to an actual organ specific internal dose (influenced by inhalation rates, lung volumes, and physical activity combined with the huge spatiotemporal variability of the external exposure) hampers the research into the health effects significantly. Integration of these complex interactions can be attempted when the µLUR methodology is extended to health research. Specific correction functions, each supplying an analytical function for designated corrections, can be investigated in smaller and more specifically designed studies. It is even an option to build µLURs to provide specific indicators that can act as parameters in a dependent µLUR. A participatory campaign tracking physical activity with ubiquitous data can be used to build a µLUR to predict the inhalation rate and heart rate as a function of the activity, spatial features, micro-environment, age, and some standard medical health related parameters. The resulting indicator, calculated at a temporal resolution similar to the exposure indicator, can become part of the dose corrections of the internal dose sensitive indicator. This requires full integration of the data, knowledge, and uncertainties across disciplines.

#### 4.5.2. Interaction between Personal Data and µLUR

The personal data and the activity specific models do not have to be independent. Personal attributes can be included in the µLUR. The in-vehicle model is an exemplary case. The actual driving forces of in-vehicle exposure are the emission specific features of the vehicles driving ahead of the monitored vehicle combined with the vehicle and user specific parameters of ventilation quality and ventilation settings. None of these driving forces can be quantified for epidemiological cohorts. Scientific experiments attempting to quantify this physical relation are interesting but fail to fulfil the requirement of applicability for health research, the main concern of Paul Lioy and Kirk Smith [1]. The choice of parameters to resolve personal exposure can be directed by potential available data on the personal and activity level of the mobile population. In other words, the attributes of the epidemiological cohorts can limit the choice of covariates in the µLUR. Of course, the attribution of the personal attributes can be extended. This might be achieved in specific scientific setups, but it will not be possible for all driving forces for large cohorts. The final aim of the models should be applicability for epidemiological and policy related solutions. Limiting the model covariates to achieve this goal is a valid option.

#### 4.5.3. Exposome Related Applications

The presented case is initiated on the long-standing issue of the potential confounding of traffic related noise and air pollution health effects. Tétreault and colleagues already stated in 2013 that the investigation of noise related health effects implements a higher spatial and temporal detail in the exposure compared to current practice in the air pollution discipline [15]. Transferring this spatiotemporal detail into the adjacent discipline fits the multidisciplinary integration and results in important synergies. In this specific case, it is even a fundamental requirement to resolve the potential mutual confounding and interactions of traffic related noise and air pollution exposure. Disentangling the health effects of noise can only be successful if the driving forces of traffic and traffic dynamics are assessed in the same spatiotemporal resolution for both disciplines. 

When this functionality is put into a larger perspective, the exposome can be translated in a wide and multidisciplinary set of Indicator Calculations, all processed with an identical set of individual properties of the subjects in the study population.

A different aspect of long-term health effects is about assessing the personal life cycle. The accumulated exposure over time is not only affected by the evolution of the emissions of the investigated indicator but also the changing personal features over time. The person factory covers this application. Each individual has a personal history of living and working habits. Each episode with potentially relevant changes in dwelling and/or work locations and the related changes in transport choice can be made available by defining different diurnal patterns, valid for a certain episode in the personal life history. The Indicator Calculation is sensitive to time, and, if the external data and the evolution over time of the indicator exposure characteristics can be quantified, an accumulative life-time exposure can be calculated.

## 5. Conclusions

Instantaneous microscopic land-use regression modeling (µLUR) is an innovative method based on data science techniques to model participatory sensing data. The µLUR models are activity specific and result in indicator prediction functions. A data workflow is presented to build µLUR models on available participatory sensing data. The data workflow is capable of extrapolating the activity specific µLURs to any mobile population.

The µLUR technique and data workflow are illustrated with existing models for the specific case of exposure to traffic related Black Carbon for different micro-environments. The micro-environment specific models are combined into an instantaneous personal exposure model for Black Carbon, covering a full diurnal pattern for each individual in a participatory sensing campaign, including an external validation. The multidisciplinary use of proxies as driving forces is the fundamental innovation in the presented case of daily exposure to Black Carbon.

The micro-environment specific models illustrate the properties and capabilities of the µLUR approach in using participatory sensing to gather data over all driving forces. Extending the instantaneous models to yearly or long-term exposure assessments is an inherent functionality of the µLUR. The increased spatiotemporal resolution enables the use of non-linear modelling techniques. The combined functionality of the µLUR and the data workflow is compatible with the exposome approach. Multiple disciplines can build multiple indicators on the same mobile population for both health research and policy support applications.

## Figures and Tables

**Table 1:** Overview of the features of the microscopic Land Use Regression (µLUR) methodology.

The Microscopic and Micro-Environment Specific Non-Linear Spatiotemporal Land-Use Regression Model (µLUR)
A µLUR should at least have the following features:
1. Low aggregation level of the measurements.
2. Detailed spatial and temporal attribution of all driving forces.
3. Measurement campaigns designed to capture the variability over all driving forces.
4. Activity and/or micro-environment specific models.
5. Non-linear modelling techniques capable of adjusting for non-linear aspects and/or saturation of the pollution indicator towards the driving forces.

## Supplementary Materials

Supplementary File 1
