# Supplementary figures and images for: Extending Participatory Sensing to Personal Exposure Using Microscopic Land Use Regression Models
Source: Int J Environ Res Public Health. 2017 May 31;14(6):586. doi: 10.3390/ijerph14060586 (PMC5486272; doi:10.3390/ijerph14060586)

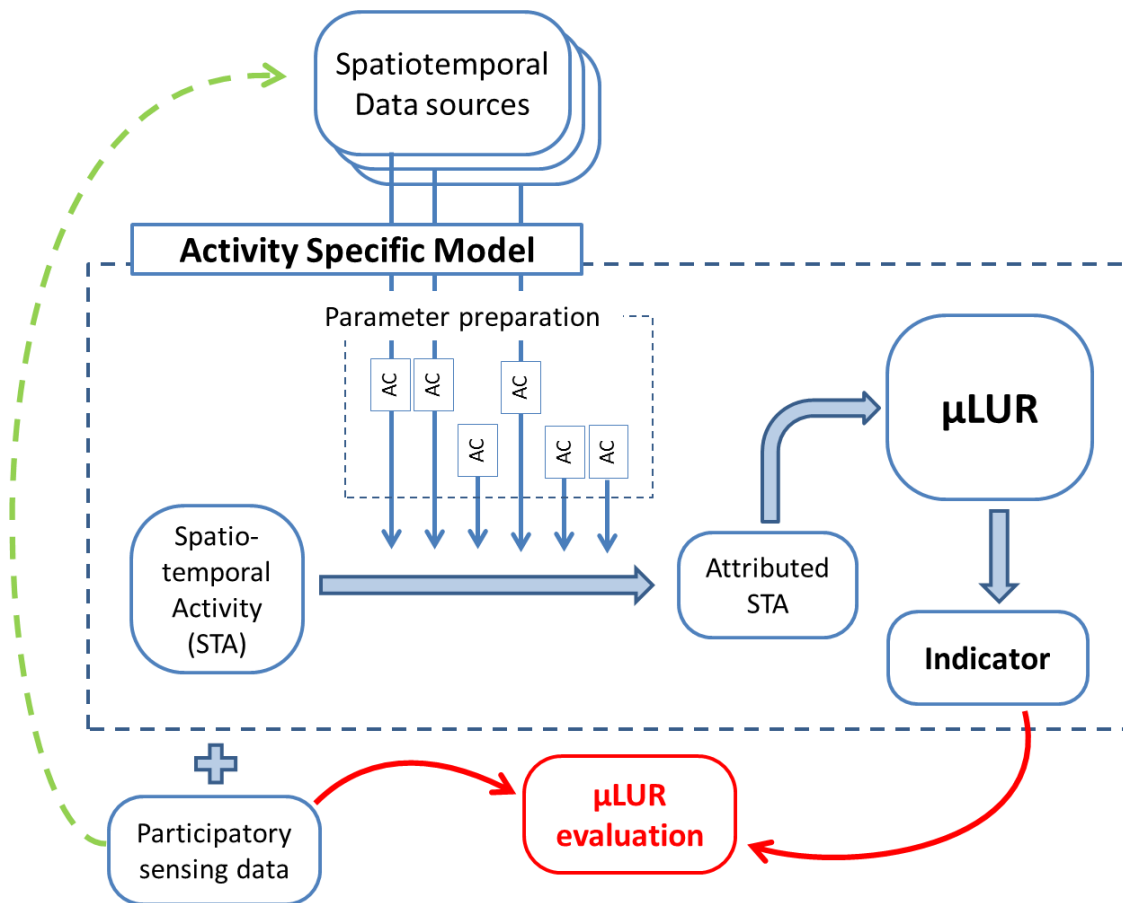

Supplement: Supplementary File 1 [file ijerph-14-00586-s001.zip › Figures/Figure_1.pdf]

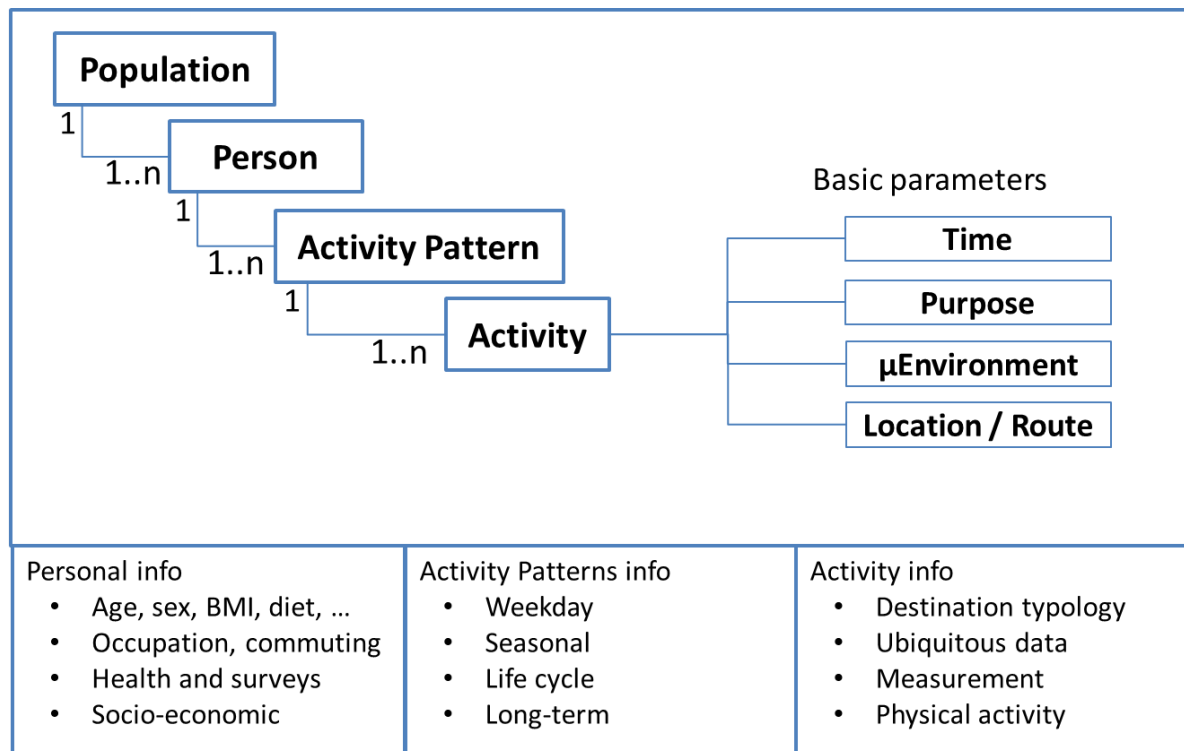

Supplement: Supplementary File 1 [file ijerph-14-00586-s001.zip › Figures/Figure_2.pdf]

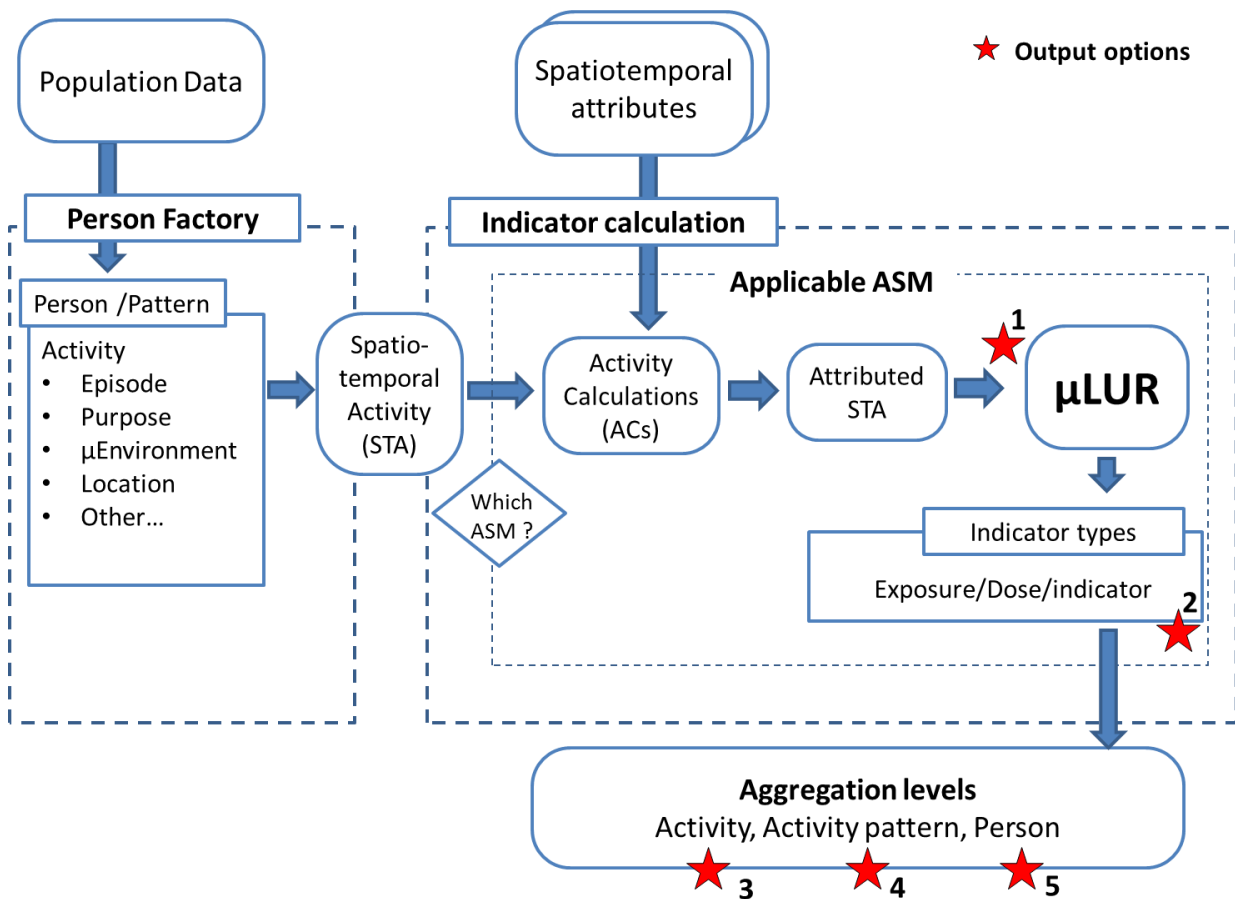

Supplement: Supplementary File 1 [file ijerph-14-00586-s001.zip › Figures/Figure_3.pdf]

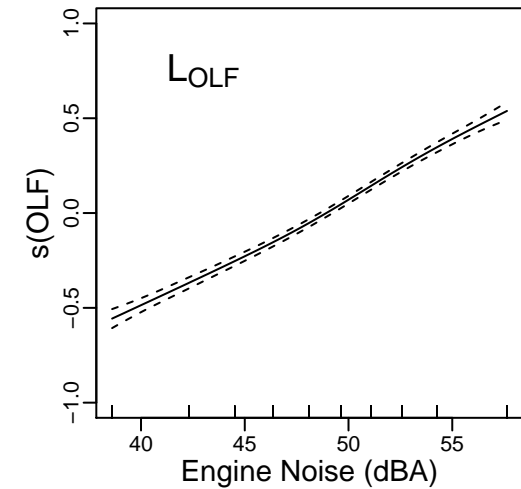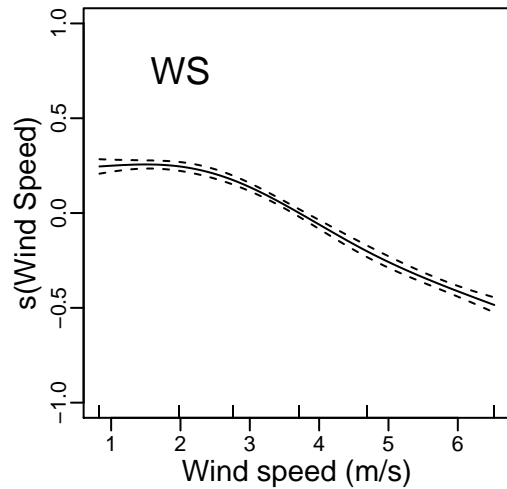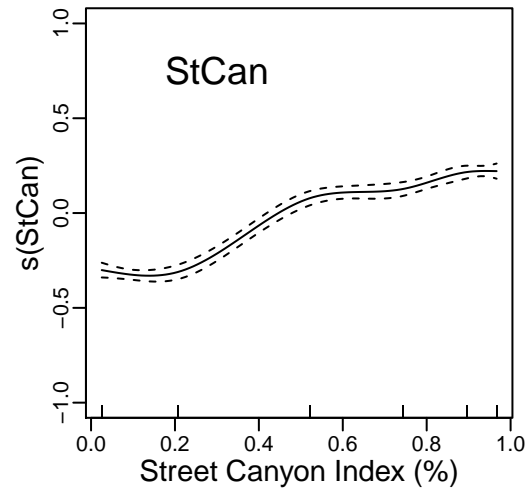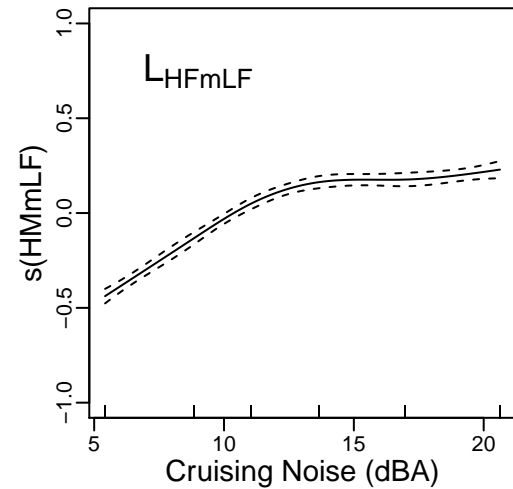

Supplement: Supplementary File 1 [file ijerph-14-00586-s001.zip › Figures/Figure_4.pdf]

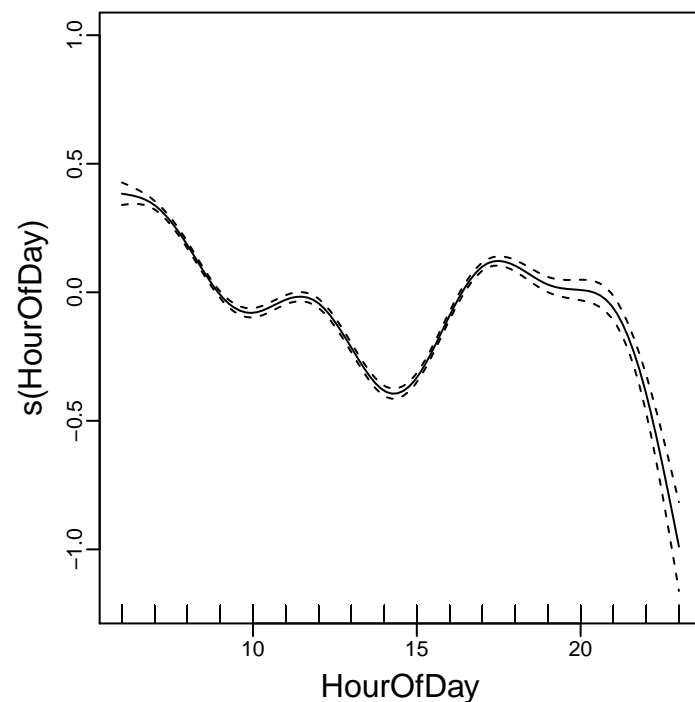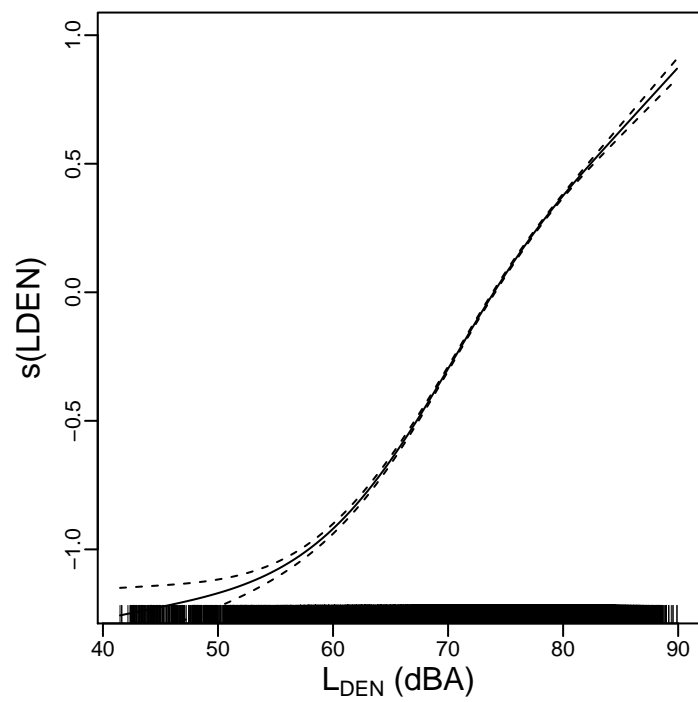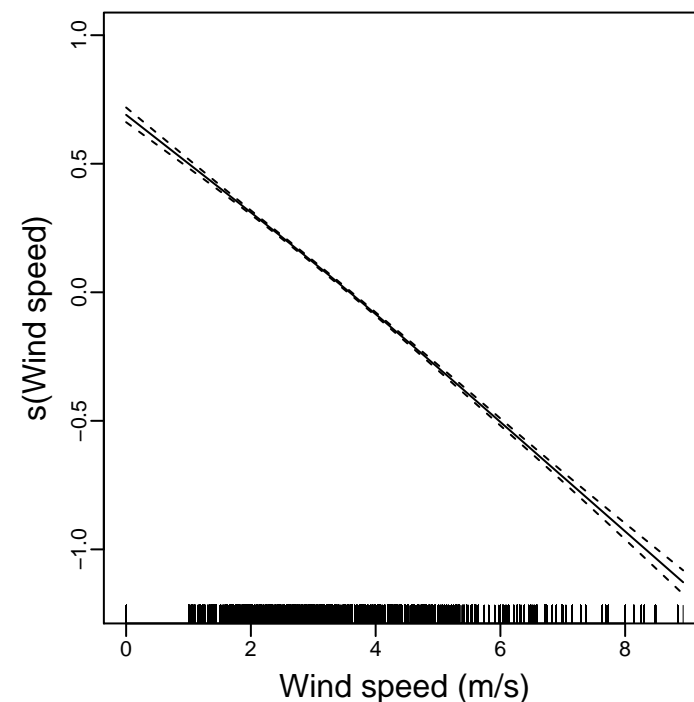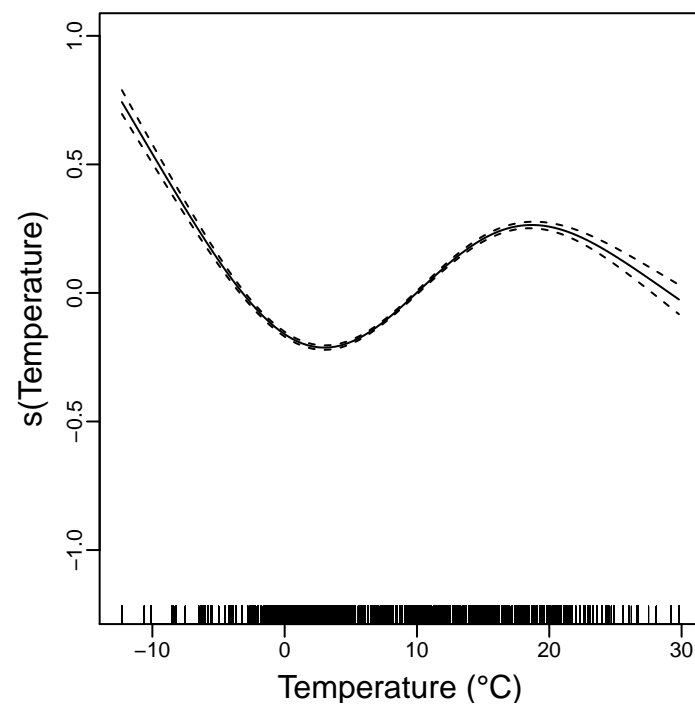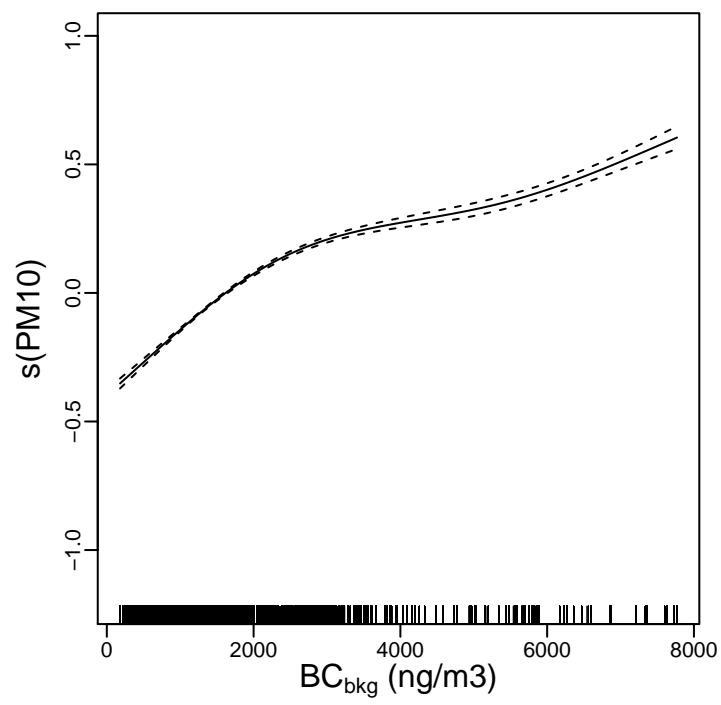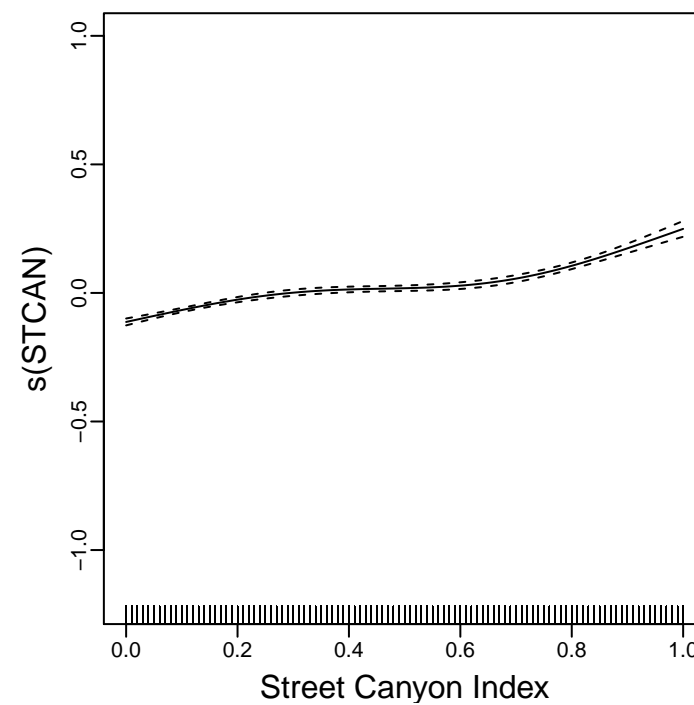

Supplement: Supplementary File 1 [file ijerph-14-00586-s001.zip › Figures/Figure_5.pdf]

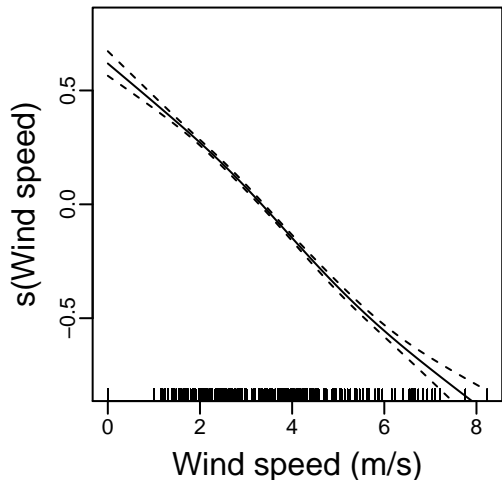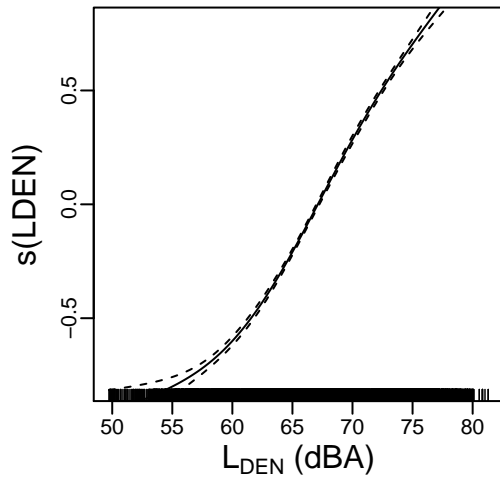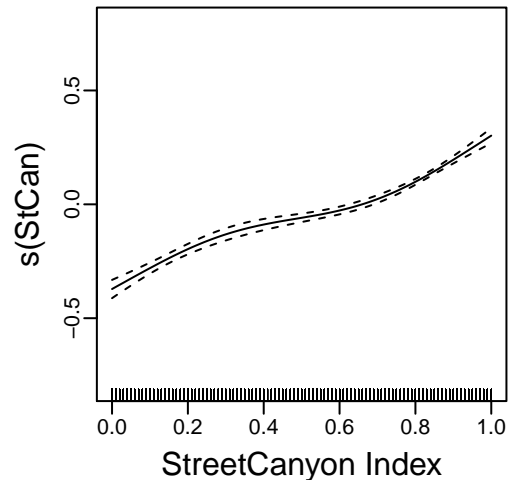

Supplement: Supplementary File 1 [file ijerph-14-00586-s001.zip › Figures/Figure_6.pdf]

## Person-Day external validation

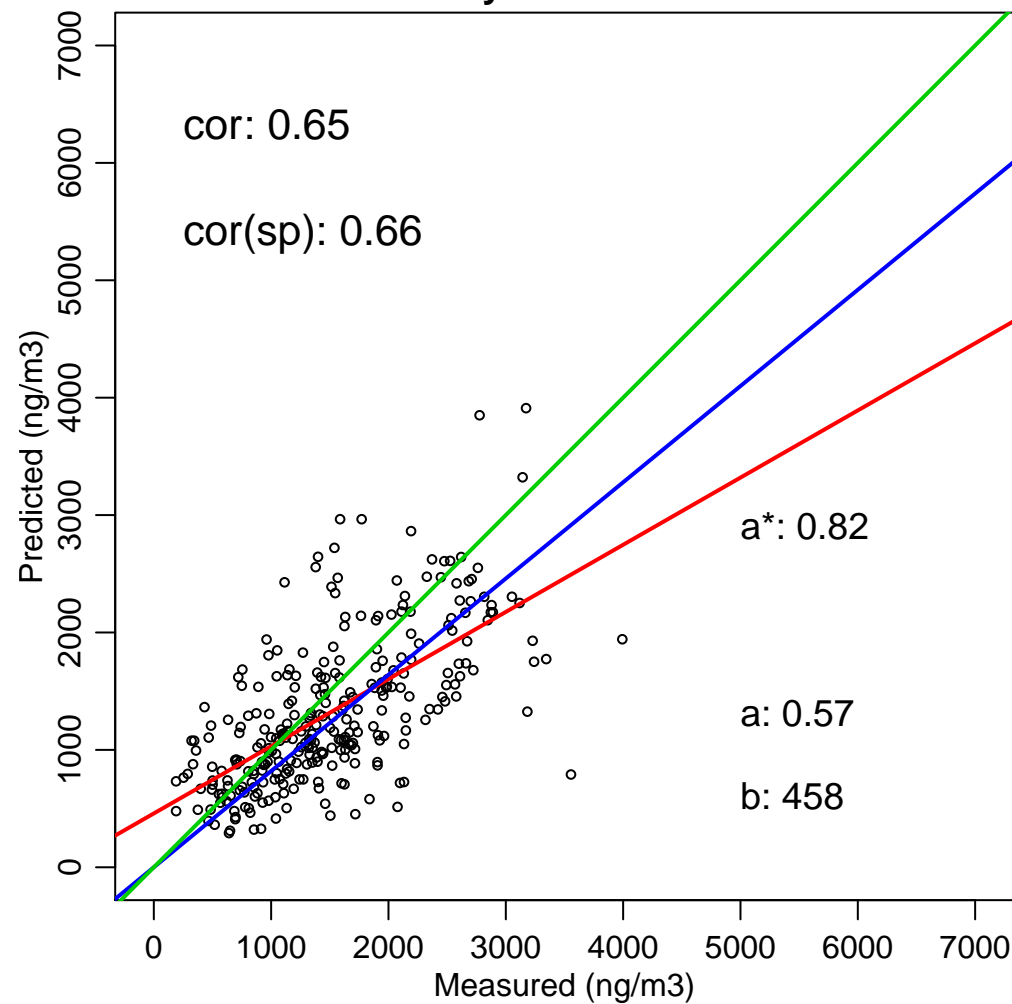

Supplement: Supplementary File 1 [file ijerph-14-00586-s001.zip › Figures/Figure_7.pdf]
